# Effectiveness of β-TriCalcium Phosphate for Alveolar Ridge Preservation: A Systematic Review

**DOI:** 10.3390/jfb17050247

**Published:** 2026-05-15

**Authors:** Vitolante Pezzella, Andrea Blasi, Leopoldo Mauriello, Giuseppe Trapanese, Elio Ramaglia, Michele Basilicata, Vincenzo Iorio-Siciliano, Luca Ramaglia

**Affiliations:** 1 Department of Periodontology, School of Dental Medicine, University of Naples Federico II, 80131 Naples, Italy; vitolante.pezzella@unina.it (V.P.); andrea.blasi@unina.it (A.B.); luca.ramaglia@unina.it (L.R.); 2 Department of Neuroscience, Reproductive Science and Dentistry, University of Naples Federico II, 80131 Naples, Italy; giuse.trapanese@studenti.unina.it; 3 Department of Clinical Medicine and Surgery, University of Naples Federico II, 80131 Naples, Italy; elioramaglia00@gmail.com; 4 UOSD Special Care Dentistry, Department of Experimental Medicine and Surgery, University of Rome Tor Vergata, 00133 Rome, Italy; michele.basilicata@ptvonline.it

**Keywords:** beta-tricalcium phosphate, bone substitutes, tooth extraction, bone regeneration, osteoconduction, cone–beam computed tomography

## Abstract

Alveolar ridge preservation (ARP) aims to reduce post-extraction bone resorption and facilitate implant placement. Among alloplastic grafts, β-tricalcium phosphate (β-TCP) is widely used due to its osteoconductive properties and complete resorbability. This systematic review evaluated the clinical effectiveness of β-TCP for ARP, focusing on ridge dimensional changes assessed by cone–beam computed tomography (CBCT). Electronic searches were performed in major scientific databases up to April 2026. Randomized controlled trials (RCTs) reporting CBCT-based dimensional outcomes after at least 4 months were included. Five RCTs met the inclusion criteria. Considerable heterogeneity was observed in biomaterial formulations, socket management, and outcome assessment. When used alone, β-TCP showed variable results, ranging from greater ridge resorption compared with xenograft to outcomes comparable with those of freeze-dried bone allograft. More consistent findings were reported when β-TCP was used in combination with other biomaterials, with outcomes generally comparable to those of deproteinized bovine bone mineral (DBBM). Overall, β-TCP may have a potential role in alveolar ridge preservation; however, evidence remains limited and heterogeneous. Differences between β-TCP alone and composite formulations should be carefully considered, and no definitive conclusions can be drawn regarding its comparative predictability versus xenografts. Further RCTs are needed to clarify its clinical effectiveness and identify optimal applications.

## 1. Introduction

Tooth extraction triggers a cascade of biological events, mediated by the local inflammatory response that follows the surgical procedure, loss of periodontal ligament vascular supply, and bundle bone remodeling, resulting in a significant three-dimensional alveolar resorption [1,2]. The most pronounced bone remodeling occurs within the first 3–6 months, after which an average horizontal reduction of 29–63% and vertical reduction of 11–22% are observed [3,4]. At 12 months following tooth extraction, the horizontal bone resorption is approximately 50%, most of which occurs in the first 3 months [5]. Moreover, the buccal plate is particularly susceptible to resorption due to its thin cortical structure and limited vascular supply; consequently, ridge remodeling is often characterized by a palatal displacement of the alveolar crest and a greater reduction in ridge height on the buccal aspect [2,6]. These changes may lead to a deformed edentulous ridge and reduced bone volumes that negatively affect prosthetically driven implant placement, often requiring additional bone augmentation procedures [7].

Alveolar ridge preservation (ARP) has therefore become a widely adopted surgical procedure in order to limit post-extraction bone resorption and improve the conditions for subsequent implant rehabilitation [8]. A systematic review and meta-analysis by Willenbacher et al. demonstrated that 20.8% of sites with unassisted socket healing required further bone augmentation, while only 9.9% of the sites that received ARP needed additional augmentation [9].

Several biomaterials have been proposed for ARP, including xenografts, allografts, and synthetic bone substitutes, frequently combined with barrier membranes or soft tissue sealing techniques [10,11].

Among these, β-tricalcium phosphate (β-TCP) represents one of the most commonly used alloplastic graft materials due to its osteoconductive properties and slow resorbability [12,13,14]. Used as a graft in bone regeneration procedures, it serves as a three-dimensional scaffold supporting new bone formation, while counteracting tissue shrinkage and ensuring sufficient structural strength [15]. Histological studies showed that β-TCP integrates well into the bone matrix and is completely resorbed after approximately 1 year, being gradually replaced by newly formed bone [16,17]. Finally, it has been proposed as a valid alternative to xenografts and allografts since it overcomes ethical and immunological issues associated with human or animal-derived grafts and can reduce production costs [18,19].

β-TCP has been used in periodontal regenerative surgery, in treating periodontal infra-bony defects around natural teeth, showing comparable results to other bone graft materials in terms of pocket depth reduction, clinical attachment level gain, and bone fill [20]. In bone regeneration, however, this alloplastic graft has been used above all in maxillary sinus lift procedures and in alveolar preservation surgeries [21,22,23].

Despite its widespread clinical use, evidence supporting β-TCP as a predictable material for alveolar ridge preservation remains limited and inconsistent. Therefore, the aim of the present systematic review was to assess the clinical effectiveness of β-TCP in alveolar ridge preservation, focusing on dimensional changes in post-extraction sites.

## 2. Materials and Methods

A systematic review was conducted in accordance with Preferred Reporting Items for Systematic reviews and Meta-Analyses (PRISMA) guidelines [24] and the PRISMA checklist was used to ensure complete reporting of the review process (Supplementary Table S3). The study protocol was prospectively registered in the International Prospective Register of Systematic Reviews (PROSPERO) under the registration number CRD420261283207.

### 2.1. Focused Question

The primary question of this systematic review was as follows: “Is β-tricalcium phosphate effective in preserving alveolar ridge volume following tooth extraction compared with spontaneous healing or alternative biomaterials?”.

The eligibility criteria were established according to the PICOS framework:

- Population (P): Adult patients who need to undergo tooth extraction;
- Interventions (I): ARP performed using β-TCP;
- Comparisons (C): Spontaneous socket healing or ARP performed with other biomaterials instead of β-TCP;
- Outcomes (O): Changes in alveolar ridge width and height assessed by CBCT after at least 4 months of follow-up;
- Study design (S): Randomized controlled trials (RCTs), non-randomized controlled clinical trials, and prospective cohort studies.

### 2.2. Selection Criteria

Studies were included in the present review in accordance with the following inclusion criteria: (1) studies performed in vivo on humans, (2) studies including adults (≥18 years old) who have undergone dental extraction and ARP, (3) studies evaluating the use of β-TCP, either alone or in combination with other biomaterials, for ARP following tooth extraction, (4) studies reporting changes in ridge height and width, assessed through CBCT measurements, after at least 4 months, and (5) RCTs, non-randomized controlled clinical trials, and prospective cohort studies. Split-mouth and parallel-arm designs were accepted, and no restrictions were applied regarding blinding methods.

Studies were excluded from the review in accordance with the following exclusion criteria: (1) animal or in vitro studies, (2) studies including patients with systemic conditions, (3) studies including extraction socket of third molars, and (4) non-comparative studies, cross-sectional studies, reviews, case series, case reports, letters, and abstracts.

### 2.3. Search Strategy and Study Selection

The following electronic databases were searched: Embase, PubMed, Scopus, and Web of Science. The last search was conducted in April 2026. In addition, reference lists of the included studies were screened to identify any additional relevant articles.

The search strategy combined keywords and free-text terms related to this topic, such as “tricalcium phosphate”, “ridge preservation” and “socket preservation”, using appropriate syntax rules for each database; all combinations using (AND, OR) were utilized to refine the search results. The full strategies are provided in Supplementary Table S1.

All retrieved studies were imported into Rayyan (Rayyan Systems Inc., Cambridge, MA, USA), a web-based and mobile platform [24], and any duplicates were identified and subsequently removed. Analysis and selection of database search results were conducted independently by two reviewers (V.P. and G.T.) in a two steps process. Titles and abstracts of all studies were screened in the first step of selection. Further screening of the full texts of the selected articles was performed during the second step of the study selection, and articles that did not match the inclusion criteria were excluded from consideration. Disagreements or uncertainties between reviewers were resolved through discussion and consultation with a third reviewer (A.B.).

### 2.4. Data Extraction

The data were independently extracted by two authors (V.P. and A.B.) using a pre-determinated spreadsheet developed in Microsoft Excel (Microsoft Corporation, Redmond, Washington, USA). Any disagreements were solved by discussion. The following data were extracted from each eligible trial: (a) general study characteristics: first author name, year of publication, country, study design, clinical setting; (b) number of patients and number of treated sites; (c) patient characteristics: male/female ratio, mean age, smoking habits, oral hygiene level; (d) extraction site characteristics: single- or multi-rooted tooth, presence or absence of bone defects; (e) interventions: type of material used for ARP in test and control groups; (f) follow-up duration; (g) outcomes: mean changes in ridge width and height, assessed through CBCT.

### 2.5. Risk of Bias Assessment

The methodological quality of the included studies was independently assessed by two reviewers (V.P. and A.B.), as a part of the data extraction process, using the Cochrane Risk of Bias 2 (RoB 2) tool for RCTs [25]. The following domains were evaluated: bias arising from the randomization process, bias due to deviations from intended interventions, bias due to missing outcome data, bias in outcome measurement, and bias in selection of the reported result. The judgments within each domain lead to an overall risk of bias classification (“low risk of bias,” “some concerns,” or “high risk of bias”). Any disagreements were resolved through discussion.

### 2.6. Certainty of the Evidence

Two review authors (V.P. and A.B.) independently assessed the certainty of the evidence for the main outcomes (changes in alveolar ridge width and height) using the GRADE approach (Grading of Recommendations Assessment, Development and Evaluation Working Group), implemented through the GRADEpro Guideline Development Tool (GRADEpro GDT; McMaster University, Hamilton, ON, Canada).

The certainty of the evidence was evaluated across five domains: risk of bias, inconsistency, indirectness, imprecision, and publication bias. Disagreements between reviewers were resolved through discussion.

The overall certainty of the evidence for each outcome was classified as high, moderate, low, or very low, according to GRADE recommendations.

### 2.7. Data Synthesis

Studies were considered eligible for synthesis if they reported dimensional changes in alveolar ridge width and/or height assessed by CBCT after at least 4 months of follow-up.

Included studies were grouped according to the type of β-TCP application: (1) pure β-TCP used alone in post-extraction sockets, (2) pure β-TCP combined with adjunctive socket management techniques (e.g., membranes or soft tissue sealing), and (3) composite β-TCP formulations (e.g., β-TCP combined with other biomaterials).

Extracted data were reported as provided in the original studies. When necessary, outcomes were qualitatively compared by considering mean changes in ridge dimensions and, where available, percentage variations. No data transformation or imputation was performed.

Results were summarized using a narrative synthesis and presented in structured tables and figures. A quantitative meta-analysis was not undertaken due to substantial clinical and methodological heterogeneity across studies, including differences in biomaterial formulations, comparator groups, socket management protocols, follow-up durations, and CBCT measurement methods.

Given the limited number of included studies, no formal quantitative subgroup or sensitivity analyses were conducted. However, a structured qualitative stratification was applied to facilitate interpretation of the results in the presence of clinical heterogeneity.

## 3. Results

### 3.1. Study Selection

**Figure 1:**
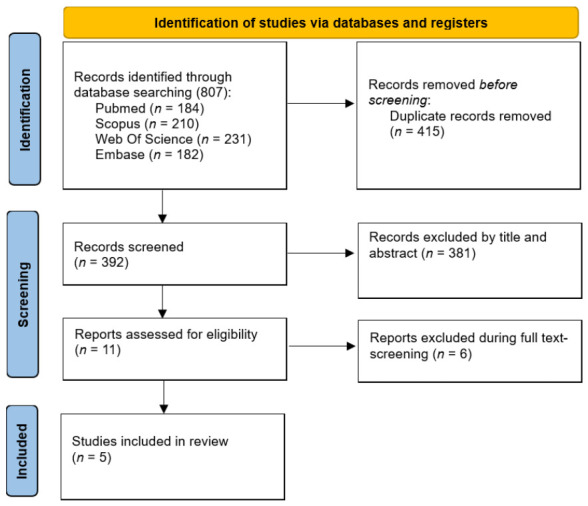
Flowchart of article retrieval process.

The study selection process is presented in Figure 1. The initial search yielded 807 articles, of which 415 were duplicates and subsequently removed. After analyzing all abstracts and titles, 11 full-text articles were shortlisted for the second phase, but 6 of these were excluded in accordance with the predetermined exclusion criteria [26,27,28,29,30,31]. Finally, a total of 5 clinical trials were identified that specifically evaluated the clinical efficacy of β-TCP used for ARP assessed through CBCT, compared either with spontaneous socket healing or with ARP performed using alternative biomaterials.

The list of excluded full-text articles with reasons is reported in Supplementary Table S2.

### 3.2. Characteristics of the Included Studies

The included studies were published between 2013 and 2025, reflecting more than a decade of accumulated evidence on the use of β-TCP in ARP [32,33,34,35,36]. All studies were conducted in university-based settings across Asia (India, China) Europe (Switzerland), and America (USA). Sample sizes ranged from 15 to 123 patients (corresponding to 30 to 123 treated sites), resulting in a total of 247 patients and 281 extraction sites included in the analysis. 

Although non-randomized controlled trials and prospective cohort studies were eligible, only RCTs met the inclusion criteria. Most studies adopted a two-arm parallel-group trials [34,35,36], one study employed a four-arm parallel design [32], while another study used a split-socket randomized design with three parallel treatment arms within the same patient [33].

### 3.3. Characteristics of Participants and Extraction Sites

Heterogeneity was observed in patient and site selection criteria. Regarding patient characteristics, two studies excluded smokers [33,34], whereas others included smokers with varying daily cigarette limits [32,35,36]; only two trials applied exclusion criteria related to oral hygiene status, assessed using plaque index (PI) or full-mouth plaque score (FMPS) [32,36]. Concerning site selection, two studies enrolled incisors, canines and premolars [32,34]; one study focused exclusively on posterior teeth (molars and premolars) [35], while the remaining studies included sites from all tooth types [33,36]. Additional variability was noted in the inclusion criteria for socket morphology: some trials included only sites with intact bony walls [33,34], others accepted sites with partial bone loss (up to 50% residual buccal cortical height) [32], while other studies did not explicitly report socket wall conditions [35,36].

### 3.4. Interventions and Comparators

Three of these clinical trials investigated the effectiveness of pure β-TCP, applied alone or in combination with a membrane [32,33,35]. The remaining two studies investigated the clinical efficacy of ARP using β-TCP in combination with other biomaterials [34,36].

### 3.5. Outcome Measures and Follow-Up

The primary outcome of this review was the prevention of alveolar ridge volume following tooth extraction, expressed as changes in alveolar ridge width and height assessed by CBCT after a minimum follow-up of 4 months. 

Heterogeneity was observed in follow-up durations among the included studies: two trials assessed outcomes at 4 months post-extraction [33,35], while the remaining studies performed tomographic evaluations after a 6-month follow-up period [32,34,36]. 

The included studies also differed in measurement protocols used to assess ridge dimensional changes. Three studies reported two mean vertical changes (for buccal and oral plates) and three mean horizontal changes: width changes were assessed at 1 mm, 3 mm, and 5 mm apical to the most coronal aspect of the alveolar crest [32,35,36]. Das et al., instead, reported two mean vertical change and three mean horizontal change: width changes were assessed at coronal, middle and apical thirds of the extraction socket [34]. Finally one study assessed only one mean vertical change and one mean horizontal change [33].

Due to substantial heterogeneity across interventions, comparators, follow-up durations, and outcome assessment methods, quantitative synthesis was not feasible and results were synthesized narratively and organized into three groups: (1) efficacy of pure β-TCP; (2) efficacy of pure β-TCP combined with adjunctive socket management techniques (e.g., membranes); and (3) efficacy composite β-TCP formulations (e.g., β-TCP combined with other biomaterials).

### 3.6. Changes in Alveolar Ridge Dimensions Using Pure β-TCP

In two studies, ARP was performed placing the pure β-TCP into the post-extraction socket without using membranes, collagen matrices, or any additional soft tissue treatment [32,35]. Both trials employed β-TCP coated with poly(lactic-co-glycolid acid) (PLGA) (easy-graft^®^, Degradable Solutions AG, Schlieren, Switzerland). The biomaterial was prepared according to manufacturer’s instructions: the graft was mixed with the N-methylpyrrolidon (NMP) liquid activator, placed and slightly compressed into the socket, and subsequently rinsed with 40 mL of sterile saline to remove the NMP.

Jung et al. compared ARP with β-TCP with spontaneous healing and with ARP performed using DBBM with 10% collagen (combined either with a collagen matrix or an autogenous free gingival graft) at 6 months of follow-up [32]. A significantly increased vertical and horizontal resorption was observed in β-TCP group (19.5–20.9%, 18.8–77.5% respectively), not only when compared with the sites treated with DBBM with 10% collagen and collagen matrix (0.1–2.6%, 1.7–17.4%) or autogenous free gingival graft (5.6–8.1%, 6.4–18.1%), but even when compared with spontaneous healing (5.5–10.2%, 8.1–43.3%). Conversely, Saito et al. compared β-TCP with freeze-dried bone allograft (FDBA) used with a rapidly absorbable collagen dressing, finding a slightly better alveolar volume preservation in the β-TCP group at 4 months of follow-up, although the differences did not reach statistical significance [35].

The main characteristics and outcomes are summarized in Table 1.

### 3.7. Changes in Alveolar Ridge Dimensions Using Pure β-TCP Combined with Adjunctive Socket Management Techniques

Joshi et al. evaluated the clinical effects of β-TCP (SyboGraf™-T Plug, Eucare Pharmaceuticals Pvt. Ltd., Chennai, Tamil Nadu, India) and autogenous tooth graft, both covered with a chorion membrane, compared with chorion membrane alone (control group) [33]. At 4 months of follow-up, sites treated with β-TCP showed reduced alveolar volume loss (height change: −1.72 ± 0.56 mm; width change: −1.45 ± 0.40 mm) compared with the control group (−2.60 ± 0.88 mm; −2.29 ± 0.40 mm) but a greater bone loss than sites treated with autogenous tooth graft (−0.28 ± 0.13 mm; −0.15 ± 0.08 mm). These differences were statistically significant (Table 2).

### 3.8. Changes in Alveolar Ridge Dimensions Using Composite β-TCP Formulation

More detailed and consistent patterns emerged when β-TCP was used in composite formulations, in which the material was combined with biologically active or structurally stabilizing components.

Das et al. performed ARP filling the post-extraction sockets with a product made with β-TCP granules coated with a matrix of highly purified collagen fibers of bovine origin (R.T.R. Cones™, Septodont, Saint-Maur-des-Fosses, France), while the control sites were treated with platelet-rich fibrin (PRF) [34]. The composite β-TCP formulation resulted in improved dimensional outcomes compared with PRF alone, including reduced horizontal resorption at coronal (−10.3% vs. −18.6%) and middle (−2.1% vs. −12%) levels, and even slight bone apposition at the apical third (4.8% vs. −15.6%); statistically significant apposition was also observed in the oral cortical plate height in the test group (11.6%), in contrast to the resorption observed in the control group (−12.43%); and regarding buccal cortical height change, sites treated with β-TCP showed better results (−12.9% vs. −15.2%), but without statistically significant differences.

Similarly, Gao et al. investigated the effects of β-TCP coating porous bovine deproteinization bone (β-TCP/PBDB) compared with DBBM (Bio-Oss^®^) [36]. In both groups, a collagen sponge was applied over the bone substitutes. At 6 months of follow-up, β-TCP/PBDB showed dimensional outcomes comparable to DBBM, with a tendency toward better vertical preservation, although differences were not statistically significant.

The main characteristics and outcomes are summarized in Table 3.

### 3.9. Risk of Bias Assessment

**Figure 2:**
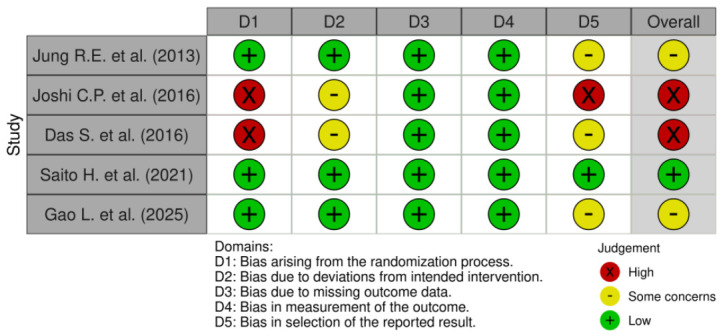
Quality assessment of the included studies according to RoB-2 tool [32,33,34,35,36].

Figure 2 summarizes the bias assessment of the included studies, using the Cochrane ROB-2 tool.

Only one trial was classified as low risk of bias [35]. Studies by Jung et al. and Gao et al. were judged as having some concerns because no explicit reference to a pre-specified statistical analysis plan or trial registration was provided [32,36]. The remaining two studies were judged to be at high risk of bias, mainly due to methodological limitations in the randomization process and concerns regarding selective reporting [33,34].

### 3.10. Certainty of the Evidence

The certainty of the evidence for the main outcomes was assessed using the GRADE approach. For both ridge width and ridge height changes, the overall certainty of evidence was rated as low.

This rating was primarily due to serious risk of bias and serious inconsistency across the included studies. Specifically, two studies were judged as having high risk of bias and two raised some concerns, reducing confidence in the estimated effects. In addition, substantial heterogeneity was observed in biomaterial formulations, comparators, follow-up durations, and CBCT measurement protocols, contributing to inconsistency in the findings. No serious concerns were identified regarding indirectness, as the included studies directly addressed the review question in terms of population, intervention, comparator, and outcomes. Similarly, imprecision was not considered serious, although the relatively small number of studies should be taken into account when interpreting the results.

The detailed GRADE assessment is reported in Table 4.

## 4. Discussion

ARP is widely performed to attenuate physiologic post-extraction ridge remodeling and to maintain adequate bone volumes for subsequent implant placement [37]. By limiting horizontal and vertical resorption, ARP may reduce the need for additional augmentation procedures and improve prosthetically driven implant positioning, particularly in sites with a thin buccal plate.

A variety of grafting materials have been proposed for ARP, including allografts, xenografts, and autologous biomaterials such as platelet concentrates or tooth-derived matrices [38,39,40,41,42]. More recently, increasing interest has been directed toward alloplastic substitutes. Among these, β-TCP has gained clinical relevance due to its osteoconductive properties, favorable biocompatibility, and complete resorbability. Histological evidence indicates that β-TCP integrates into the bone matrix and is progressively replaced by newly formed bone over time, providing a temporary scaffold for socket healing.

In the present review, dimensional outcomes were specifically assessed using CBCT, as multiplanar reconstructions enable standardized three-dimensional evaluation of ridge width and height changes, offering greater accuracy and reproducibility compared with conventional clinical measurements [43].

Five randomized controlled trials published between 2013 and 2025 were included. Overall, the findings suggest that β-TCP may have a potential role in alveolar ridge preservation; however, its dimensional effectiveness remains inconsistent and appears strongly influenced by biomaterial formulation, adjunctive socket management, and the comparator graft material.

When β-TCP was used alone, the included trials reported highly variable dimensional outcomes. Jung et al. observed significantly greater horizontal and vertical ridge resorption in sockets treated with PLGA-coated β-TCP compared not only with sites preserved using DBBM combined with collagen matrices or soft tissue grafting, but even when compared with spontaneous healing [32]. Nevertheless, these unfavorable findings should be interpreted with caution, since β-TCP was placed 1–2 mm below the bone crest without any adjunctive soft tissue sealing, whereas DBBM groups received graft at the bone level together with additional procedures, such as a collagen matrix or an autogenous soft tissue punch graft. Moreover, the β-TCP arm was the only group showing a mean buccal cortical thickness below 1 mm: although differences between groups are not statistically significant, the reduced buccal cortical thickness observed in the β-TCP group may have contributed to the greater dimensional changes reported.

In contrast, Saito et al. reported comparable dimensional changes between β-TCP and FDBA, with no statistically significant differences at 4 months [35]. Similarly, Joshi et al. found that β-TCP combined with a chorion membrane resulted in significantly reduced ridge height and width loss compared with membrane alone, although outcomes remained inferior to autogenous tooth-derived grafts [33]. Taken together, these results indicate that β-TCP may offer benefits over unassisted healing or soft tissue coverage alone, but its predictability appears inconsistent when compared with other established grafting materials.

More consistent outcomes were observed when β-TCP was applied in combination with other biomaterials. Das et al. demonstrated superior ridge preservation using β-TCP-C1 compared with PRF, including reduced horizontal resorption at multiple socket levels and even slight bone apposition in the apical third [34]. Likewise, Gao et al. reported comparable dimensional changes between β-TCP-coated bovine deproteinized bone and DBBM at 6 months, suggesting that composite formulations may enhance space maintenance and modulate resorption dynamics [36].

**Figure 3:**
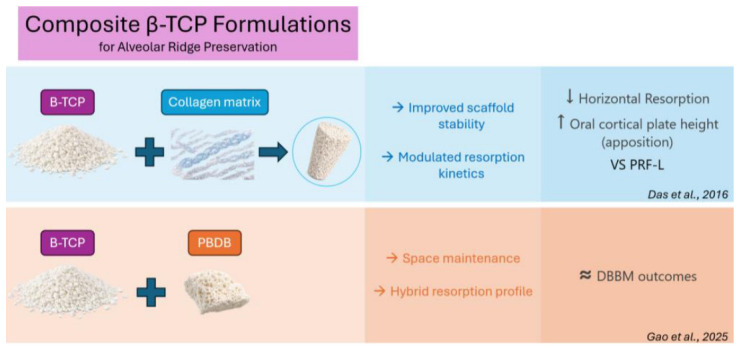
Effects of composite β-TCP formulations on alveolar ridge dimensional changes [34,36].

Overall, these findings suggest that the clinical performance of β-TCP in ARP may be significantly influenced by its formulation. In particular, more predictable dimensional preservation may be achieved when β-TCP is incorporated into biomaterials that provide additional scaffold stability or modulate resorption kinetics, compared with its use alone (Figure 3). However, the scarcity of controlled trials precludes robust conclusions.

From a clinical perspective, β-TCP may represent a potential alloplastic option for ARP, particularly when xenogeneic or allogeneic grafts are not indicated or are declined by patients. Nevertheless, dimensional outcomes may be less predictable than those achieved with slow-resorbing substitutes such as DBBM, especially in esthetic areas or in sockets with compromised buccal plates, where maximal ridge stability is required.

Several limitations of this review should be acknowledged. The available evidence is based on only five randomized controlled trials, most with relatively small sample sizes. Considerable heterogeneity was observed in socket morphology, smoking status, graft formulations (pure β-TCP versus composite materials), use of barrier membranes or soft tissue sealing techniques, and follow-up duration (4–6 months). In addition, CBCT measurement protocols were not fully standardized across studies, with differences in reference points and assessment levels, further reducing comparability. These limitations reduce the strength and generalizability of the conclusions.

Finally, another limitation of this review is the exclusive focus on CBCT-based dimensional outcomes. Although these provide objective and standardized measurements, they do not capture other relevant endpoints such as histologic bone quality, implant feasibility, need for additional augmentation, complications, or patient-reported outcomes. This may limit the clinical translatability of the findings.

Therefore, future research should include well-designed multicenter RCTs with larger sample sizes, longer follow-up periods, and standardized CBCT outcome protocols. Stratification according to socket phenotype and buccal wall integrity may further improve clinical applicability. Moreover, additional studies are warranted to clarify whether composite formulations of β-TCP can provide improved scaffold stability and more predictable ridge preservation outcomes.

## 5. Conclusions

Within the limitations of the available evidence, the effectiveness of β-TCP for alveolar ridge preservation remains uncertain, as the included studies showed heterogeneous and sometimes inconsistent findings compared with unassisted healing and other grafting materials. Reported outcomes appear variable across clinical protocols and may be influenced by biomaterial formulation and adjunctive socket management.

Differences between β-TCP used alone and in combination with other biomaterials should be carefully considered, as more consistent outcomes have been reported with composite formulations. At present, no definitive conclusions can be drawn regarding its comparative effectiveness or predictability relative to other grafting materials.

The heterogeneity in clinical outcomes may be partly explained by the biological behavior of β-TCP: as a fully resorbable osteoconductive graft, β-TCP is progressively replaced by newly formed bone; however, faster resorption may compromise early scaffold stability, particularly in sockets prone to buccal collapse. Further well-designed studies are needed to clarify these aspects and to better define its clinical indications.

## Figures and Tables

**Table 1:** Characteristics and outcomes of trials evaluating ARP with pure β-TCP.

Authors (Year)—Country	Study Design	Setting	Sample Size	Patient Selection	Site Selection	Treatment (Management of the Post-Extraction Socket)	Follow-Up	Results
Jung R.E. et al. (2013)—Switzerland [32]	RCT Four parallel arms	University	40 patients (M/F n.d.) Mean age: 55.3 ± 14.7 years 40 sites	FMBS ≤ 20% PI ≤ 20% ≤20 cigarettes/day	Maxillary and mandibular incisors, canines and premolars At least 50% of the buccal bone height present	**Test group 1**: ARP with β-TCP **Test group 2**: ARP with DBBM with 10% collagen + collagen matrix **Test group 3**: ARP with DBBM with 10% collagen + autogenous free gingival graft **Control group**: Spontaneous healing	6 months	**Test group 1:** Buccal plate height change: −2.0 ± 2.4 mm Oral plate height change: −1.7 ± 0.6 mm Ridge width change 1 mm below the ridge: −6.1 ± 2.5 mm Ridge width change 3 mm below the ridge: −3.1 ± 1.6 mm Ridge width change 5 mm below the ridge: −5.7 ± 3.0 mm **Test group 2:** Buccal plate height change: 0 ± 1.2 mm Oral plate height change: −0.4 ± 1.4 mm Ridge width change 1 mm below the ridge: −1.2 ± 0.8 mm Ridge width change 3 mm below the ridge: −0.6 ± 0.6 mm Ridge width change 5 mm below the ridge: −0.1 ± 0.2 mm **Test group 3:** Buccal plate height change: 1.2 ± 2.9 mm Oral plate height change: 0.3 ± 1.1 mm Ridge width change 1 mm below the ridge: −1.4 ± 1.0 mm Ridge width change 3 mm below the ridge: −0.6 ± 0.5 mm Ridge width change 5 mm below the ridge: −0.6 ± 0.9 mm **Control group:** Buccal plate height change: −0.5 ± 0.9 mm Oral plate height change: −0.6 ± 0.6 mm Ridge width change 1 mm below the ridge: −3.3 ± 2.0 mm Ridge width change 3 mm below the ridge: −1.7 ± 0.8 mm Ridge width change 5 mm below the ridge: −0.8 ± 0.5 mm
Saito H. et al. (2021)—USA [35]	RCT Two parallel arms	University	43 patients (M/F n.d.) Mean age: n.d. 43 sites	≤10 cigarettes/day	Maxillary and mandibular premolars and molars Absence of bone dehiscence or fenestration	**Test group 1**: ARP with β-TCP **Test group 2**: ARP with freeze-dried bone allograft + rapidly absorbable collagen dressing	4 months	**Test group 1:** Buccal plate height change: −0.53 ± 1.24 mm Oral plate height change: −0.24 ± 1.08 mm Ridge width change 1 mm below the ridge: −1.26 ± 1.32 mm Ridge width change 3 mm below the ridge: −0.61 ± 0.92 mm Ridge width change 5 mm below the ridge: −0.29 ± 0.56 mm **Test group 2:** Buccal plate height change: −0.57 ± 1.44 mm Oral plate height change: −0.85 ± 1.00 mm Ridge width change 1 mm below the ridge: −1.28 ± 1.73 mm Ridge width change 3 mm below the ridge: −0.68 ± 1.59 mm Ridge width change 5 mm below the ridge: −0.56 ± 0.75 mm

n.d.: not disclosed.

**Table 2:** Characteristics and outcomes of trials evaluating ARP with pure β-TCP combined with adjunctive socket management techniques.

Authors (Year)—Country	Study Design	Setting	Sample Size	Patient Selection	Site Selection	Treatment (Management of the Post-Extraction Socket)	Follow-Up	Results
Joshi C.P. et al. (2016)—India [33]	RCT Split-socket, three-arm within-patient	University	15 patients (9M/6F) Mean age: 35.6 ± 5.7 years 45 sites (three for patient)	No smokers	Maxillary and mandibular incisors, canines, premolars and molars Intact alveolar walls	**Test group 1**: ARP with β-TCP + chorion membrane **Test group 2**: ARP with autogenous tooth graft + chorion membrane **Control group**: Chorion membrane	4 months	**Test group 1:** Ridge height change: −1.72 ± 0.56 mm Ridge width change: −1.45 ± 0.40 mm **Test group 2:** Ridge height change: −0.28 ± 0.13 mm Ridge width change: −0.15 ± 0.08 mm **Control:** Ridge height change: −2.60 ± 0.88 mm Ridge width change: −2.29 ± 0.40 mm

**Table 3:** Characteristics and outcomes of trials evaluating ARP with composite β-TCP formulation.

Authors (Year)—Country	Study Design	Setting	Sample Size	Patient Selection	Site Selection	Treatment (Management of the Post-Extraction Socket)	Follow-Up	Results
Das S. et al. (2016)—India [34]	RCT Two parallel arms	University	26 patients (13M/13F) Mean age: 31.22 ± 8.51 years 30 sites	No smokers	Maxillary and mandibular incisors, canines and premolars Intact bone walls	**Test group 1**: ARP with β-TCP-C1 **Test group 2**: Filling the post-extraction socket with PRF	6 months	**Test group 1:** Buccal plate height change: −0.99 mm Oral plate height change: 0.94 mm Ridge width change at coronal third: −0.86 mm Ridge width change at middle third: −0.18 mm Ridge width change at apical third: 0.36 mm **Test group 2:** Buccal plate height change: −1.55 mm Oral plate height change: −1.26 mm Ridge width change at coronal third: −1.52 mm Ridge width change at middle third: −1.02 mm Ridge width change at apical third: −1.43 mm
Gao L. et al. (2025)—China [36]	RCT Two parallel arms	University	123 patients (56M/67F) Mean age: 46.51 years 123 sites	FMBS < 20% PI < Grade I ≤15 cigarettes/day	Maxillary and mandibular incisors, canines, premolars and molars	**Test group 1**: ARP with β-TCP/PBPB + collagen sponge **Test group 2**: ARP with DBBM + collagen sponge	6 months	**Test group 1:** Buccal ridge height change: −0.75 ± 1.96 mm Oral ridge height change: −0.95 ± 1.96 mm Ridge width change 1 mm below the ridge: −1.27 ± 1.32 mm Ridge width change 3 mm below the ridge: −0.89 ± 1.31 mm Ridge width change 5 mm below the ridge: −0.63 ± 1.37 mm **Test group 2:** Buccal ridge height change: −1.01 ± 2.44 mm Oral ridge height change: −0.99 ± 2.13 mm Ridge width change 1 mm below the ridge: −1.12 ± 1.65 mm Ridge width change 3 mm below the ridge: −0.55 ± 1.41 mm Ridge width change 5 mm below the ridge: −0.56 ± 1.32 mm

**Table 4:** Summary of findings of GRADE assessment.

Patients or population: Adult patients undergoing tooth extraction and ARP procedure Setting: University Intervention: ARP performed using β-TCP Comparison: Spontaneous healing or ARP performed using alternative biomaterials instead β-TCP						
**Outcome**	**Number of Participants (Studies)**	**Risk of Bias**	**Inconsistency**	**Indirectness**	**Imprecision**	**Overall Certainty of Evidence**
Ridge width change	277 (5 studies)	Serious ^1^	Serious ^2^	Not serious	Not serious	⨁⨁◯◯ Low
Ridge height change	277 (5 studies)	Serious ^1^	Serious ^2^	Not serious	Not serious	⨁⨁◯◯ Low

^1^ Downgraded one level because two studies were judged at high risk of bias and two studies raised some concerns; ^2^ downgraded due to heterogeneity in biomaterials, comparators, and measurement protocols.

## Data Availability

The original contributions presented in this study are included in the article/Supplementary Materials. Further inquiries can be directed to the corresponding authors.

## Supplementary Materials

The following supporting information can be downloaded at https://www.mdpi.com/article/10.3390/jfb17050247/s1. Table S1: Full electronic search strategies used for each database; Table S2: Full Reference List of Excluded Studies after full-text assessment with reasons; Table S3: PRISMA checklist.

## Abbreviations

The following abbreviations are used in this manuscript:

ARP	Alveolar ridge preservation
β-TCP	β-tricalcium phosphate
CBCT	Cone–beam computed tomography
RCT	Randomized controlled study
DBBM	Deproteinized bovine bone mineral
PI	Plaque index
FMPS	Full-mouth plaque score
PLGA	Poly(lactic-co-glycolid acid)
NMP	N-methylpyrrolidon
FDBA	Freeze-dried bone allograft
PRF	Platelet-rich fibrin
PBDB	Porous bovine deproteinization bone
