# Supplementary material for: Effectiveness of β-TriCalcium Phosphate for Alveolar Ridge Preservation: A Systematic Review
Source: J Funct Biomater. 2026 May 15;17(5):247. doi: 10.3390/jfb17050247 (PMC13207125; doi:10.3390/jfb17050247)
Supplement: Supplementary file 1 [file jfb-17-00247-s001.zip › Supplementary Table S1, S2.pdf]

Supplementary Table S1. Full electronic search strategies used for each database.

Database	Search string
Embase	('tricalcium phosphate':ti,ab,kw OR 'beta tricalcium phosphate':ti,ab,kw OR 'β-tcp':ti,ab,kw OR 'beta tcp':ti,ab,kw OR 'β tricalcium phosphate':ti,ab,kw) AND ('alveolar ridge preservation':ti,ab,kw OR 'ridge preservation':ti,ab,kw OR 'socket preservation':ti,ab,kw OR 'alveolar preservation':ti,ab,kw OR 'post-extraction socket':ti,ab,kw OR 'extraction socket':ti,ab,kw OR 'tooth extraction':ti,ab,kw OR 'alveolar bone resorption':ti,ab,kw OR 'ridge augmentation':ti,ab,kw)
PubMed	((("tricalcium phosphate"[Supplementary Concept] OR "tricalcium phosphate"[All Fields]) AND (("ridge"[All Fields] OR "ridge s"[All Fields] OR "ridged"[All Fields] OR "ridges"[All Fields] OR "ridging"[All Fields]) AND ("preservation, biological"[MeSH Terms] OR ("preservation"[All Fields] AND "biological"[All Fields]) OR "biological preservation"[All Fields] OR "preservation"[All Fields] OR "preserved"[All Fields] OR "preservations"[All Fields] OR "preserve"[All Fields] OR "preserves"[All Fields] OR "preserving"[All Fields])))) OR ((("tricalcium phosphate"[Supplementary Concept] OR "tricalcium phosphate"[All Fields]) AND (("periodontal ligament"[MeSH Terms] OR ("periodontal"[All Fields] AND "ligament"[All Fields]) OR "periodontal ligament"[All Fields] OR "socket"[All Fields] OR "sockets"[All Fields]) AND ("preservation, biological"[MeSH Terms] OR ("preservation"[All Fields] AND "biological"[All Fields]) OR "biological preservation"[All Fields] OR "preservation"[All Fields] OR "preserved"[All Fields] OR "preservations"[All Fields] OR "preserve"[All Fields] OR "preserves"[All Fields] OR "preserving"[All Fields])))) OR (("tricalcium phosphate"[Title/Abstract] OR "beta tricalcium phosphate"[Title/Abstract] OR "β-TCP"[Title/Abstract] OR "beta TCP"[Title/Abstract] OR "β tricalcium phosphate"[Title/Abstract]) AND ("alveolar ridge preservation"[Title/Abstract] OR "ridge preservation"[Title/Abstract] OR "socket preservation"[Title/Abstract] OR "alveolar preservation"[Title/Abstract] OR "post-extraction socket"[Title/Abstract] OR "extraction socket"[Title/Abstract] OR "tooth extraction"[Title/Abstract] OR "alveolar bone resorption"[Title/Abstract] OR "ridge augmentation"[Title/Abstract]))
Scopus	(TITLE-ABS-KEY ("tricalcium phosphate" OR "beta tricalcium phosphate" OR "β-TCP" OR "beta TCP")) AND (TITLE-ABS-KEY ("alveolar ridge preservation" OR "ridge preservation" OR "socket preservation" OR "alveolar preservation" OR "post-extraction socket" OR "extraction socket" OR "tooth extraction" OR "alveolar bone resorption"))
Web Of Science	TS=(("tricalcium phosphate" OR "beta tricalcium phosphate" OR "β-TCP" OR "beta TCP") AND ("alveolar ridge preservation" OR "ridge preservation" OR "socket preservation" OR "alveolar preservation" OR "post-extraction socket" OR "extraction socket" OR "tooth extraction" OR "alveolar bone resorption"))

Supplementary Table S2. Full reference list of excluded studies after full-text assessment with reasons.

Authors (year)—Country	Study design	Treatment (management of the post-extraction socket)	Reason for exclusion
Mayer Y. Et al. (2016)—Israel [1]	RCT<br><br>Two parallel groups	**Test group:**<br>ARP with B-TCP + HA + BCS<br><br>**Control group:**<br>Spontaneous healing	Incompatible measurement method (clinical assessment instead of CBCT-based 3D analysis)
Alharissy M. et al. (2018)—Syria [2]	RCT<br><br>Two parallel groups	**Test group:**<br>ARP with B-TCP<br><br>**Control group:**<br>ARP with Cockle-shell	Absence of a conventional control group
Ahmed E.A.E. et al. (2019)—Egypt [3]	RCT<br><br>Two parallel arms	**Test group 1**:<br>ARP with B-TCP + Collagen membrane<br><br>**Test group 2**:<br>ARP with BCP + Collagen membrane	Incompatible measurement method (2D periapical radiographs instead of CBCT-based 3D analysis)
Mendoza-Azpur G. et al. (2019)—Peru [4]	RCT<br><br>Two parallel arms	**Test group 1**:<br>ARP with B-TCP + Cross-linked collagen membrane<br><br>**Test group 2**:<br>Filling the post-extraction socket with PRF-L	No extractable data for primary outcome
Han J.J. et al. (2021)—China [5]	RCT<br><br>Two parallel arms	**Test group 1**:<br>ARP with rhBMP-2/B-TCP<br><br>**Test group 2**:<br>ARP with B-TCP	Both study groups received β-TCP-based grafting materials
Sun Y. et al. (2023)—Netherlands and China [6]	RCT<br><br>Three parallel arms	**Test group 1**:<br>ARP with B-TCP + Bilayer collagen membrane<br><br>**Test group 2**:<br>ARP with rhBMP-2/BioCaP/βTCP + Bilayer collagen membrane<br><br>**Control group:**<br>Bilayer collagen membrane	Incompatible outcome measure

RCT: randomized controlled trial; ARP: alveolar ridge preservation; B-TCP: β-tricalcium phosphate; HA: hydroxyapatite; BCS: biphasic calcium sulphate; BCP: biphasic calcium phosphate; PRF-L: platelet-rich fibrin; rhBMP-2: recombinant human bone morphogenetic protein-2; BioCaP: Calcium phosphate bioceramics.

References

1. Mayer, Y.; Zigdon-Giladi, H.; Machtei, E.E. Ridge Preservation Using Composite Alloplastic Materials: A Randomized Control Clinical and Histological Study in Humans. *Clin. Implant. Dent. Relat. Res.* **2016**, *18*, 1163–1170, https://doi.org/10.1111/cid.12415.
2. Alharissy, M.; AbouSulaiman, A.; Manadili, A.; Dayoub, S. Radiographic Alternations in Alveolar Bone Dimensions Following Socket Preservation Using Two Bone Substitutes. *Journal of International Dental and Medical Research* **2018**, *11*, 906–910.

3. Ahmed, E.A.E.; Hussien, M.M.; Hasanneen, A.M. Clinical and Radiographic Evaluation of Alveolar Bone Changes Following Ridge Preservation with Two Different Biomaterials. *Ain Shams Dental Journal* **2019**, *16*, 87–94, https://doi.org/10.21608/asdj.2019.164509.
4. Mendoza-Azpur, G.; Olaechea, A.; Padial-Molina, M.; Gutiérrez-Garrido, L.; O'Valle, F.; Mesa, F.; Galindo-Moreno, P. Composite Alloplastic Biomaterial vs. Autologous Platelet-Rich Fibrin in Ridge Preservation. J. Clin. Med. 2019, 8, 223. https://doi.org/10.3390/jcm8020223.
5. Han, J.J.; Chang, A.R.; Ahn, J.; Jung, S.; Hong, J.; Oh, H-K.; Hwang, S.J. Efficacy and Safety of RhBMP/β-TCP in Alveolar Ridge Preservation: A Multicenter, Randomized, Open-Label, Comparative, Investigator-Blinded Clinical Trial. *Maxillofac. Plast. Reconstr. Surg.* **2021**, *43*, 1–14, https://doi.org/10.1186/s40902-021-00328-0.
6. Sun, Y.; Xu, C.; Wang, M.; Wei, L.; Pieterse, H.; Wu, Y.; Liu, Y. Radiographic and Histological Evaluation of Bone Formation Induced by RhBMP-2-Incorporated Biomimetic Calcium Phosphate Material in Clinical Alveolar Sockets Preservation. *Int. J. Implant. Dent.* **2023**, *9*, 1–11, https://doi.org/10.1186/s40729-023-00491-1.
